# Supplementary material for: High‐frequency longitudinal white matter diffusion‐ and myelin‐based MRI database: Reliability and variability
Source: Hum Brain Mapp. 2023 Apr 17;44(9):3758–80. doi: 10.1002/hbm.26310 (PMC10203793; doi:10.1002/hbm.26310)
Supplement: Supplementary file 1 — Data S1: Supporting Information [file HBM-44-3758-s001.pdf]

High-frequency longitudinal white matter diffusion- & myelin-based MRI database: reliability and variability

Manon Edde[1,2], Guillaume Theaud[2], Matthieu Dumont[2], Antoine Théberge[1,3], Alex Valcourt-Caron[1], Guillaume Gilbert[4], Jean-Christophe Houde[2], Loika Maltais[2], François Rheault[5], Federico Spagnolo[6], Muhamed Barakovic[6], Stefano Magon[6*], and Maxime Descoteaux[1,2*]

Affiliation

[1]Sherbrooke Connectivity Imaging Lab (SCIL), Université de Sherbrooke, Sherbrooke, QC, Canada

[2] Imeka Solutions, Inc., Sherbrooke, QC, Canada.

[3] Videos & Images Theory and Analytics Laboratory (VITAL), Université de Sherbrooke, Sherbrooke, QC, Canada

[4] MR Clinical Science, Philips Healthcare Canada, Mississauga, Ontario, Canada

[5]Medical Imaging and Neuroinformatic (MINi) Lab, Université de Sherbrooke, Sherbrooke, QC, Canada

[6] Pharma Research and Early Development, Neuroscience and Rare Diseases Roche Innovation Center Basel, F. Hoffmann-La Roche Ltd., Basel, Switzerland

*Co-senior authors

1. MRI acquisition

There were no upgrades or changes in MR scanner hardware or software during the period of this study. Procedures were standardized to limit session variability. The positioning of the head followed the standard operating procedure and the definition of the field of view (FOV) during the acquisition followed an explicit procedure; both were checked by the first author. Subject movements and evidence of motion artifacts were checked during the acquisition. No subject was excluded during acquisition, and MRI images had to be repeated in one subject during a session due to artifacts.

Table 1. MRI protocol parameters.

Parameters \ Sequences	T1	DWI	Reverse B0	ihMT	T1 ihMT
**Phase-encoding direction***	RL	PA	AP	RL	RL
**Technique – Fast imaging method**	FFE – TFE	SE – EPI	SE – EPI	FFE – EPI	FFE – EPI
**Duration**	4 min 20s	9 min 20s	14 s	6 min 04s	13 s
**TR (ms)**	7.9	4800	4800	112	20
**TE (ms)**	3.5	92	92	3.6 (Δ=6)	3.6 (Δ=6)
**TI (ms)**	950				
**Flip Angle (degree)**	8	90	90	15	30
**FOV (mm)**	224x224	224 x 224	224x224	224x224	224x224
**Slices**	150	66	66	65	65
**Voxel size (mm)**	1x1x1	2x2x2	2x2x2	2x2x2	2x2x2
**n b0, b-value (n directions)**		7, 300 (8), 1000 (32),2000 (60)			
**MT Saturation pulse**				10 Hann pulses of 0.9 ms duration with 1.5 ms interval	
**Frequency offset of +/-**				7000 Hz	
**Echoes – Echo spacing**				3 – 6 ms	

*The directions are specified in the standard way in coordinates of the patient (LPH).

An example of bvec and bval file can be downloaded here: https://high-frequency-mri-database-supplementary.readthedocs.io/en/latest/data/data_description.html#mri-acquisition-parameters

2. Inhomogeneous Magnetization Transfer imaging (ihMT)

Acquisition The multi-echo segmented-EPI sequence used for ihMT was using an EPI factor of 5 and allowed the acquisition and reconstruction of 3 echoes (echo spacing = 6.0 ms), for a total number of k-space lines per excitation of 15. However, only data from the first of the three echoes (TE = 3.6 ms) was used for our analysis. It is important to note that the other 2 echoes could be acquired without any scan time penalty, given that the minimum repetition time (TR) was SAR-limited (see Figure 1). The root means square B1 (B1rms) are computed using the equations from the Soustelle et al., 2022:

- the B1rms over the sequence TR was 2.7 uT (equation 2).
- the B1rms over one saturation pulse was 9.2 uT (equation 3).

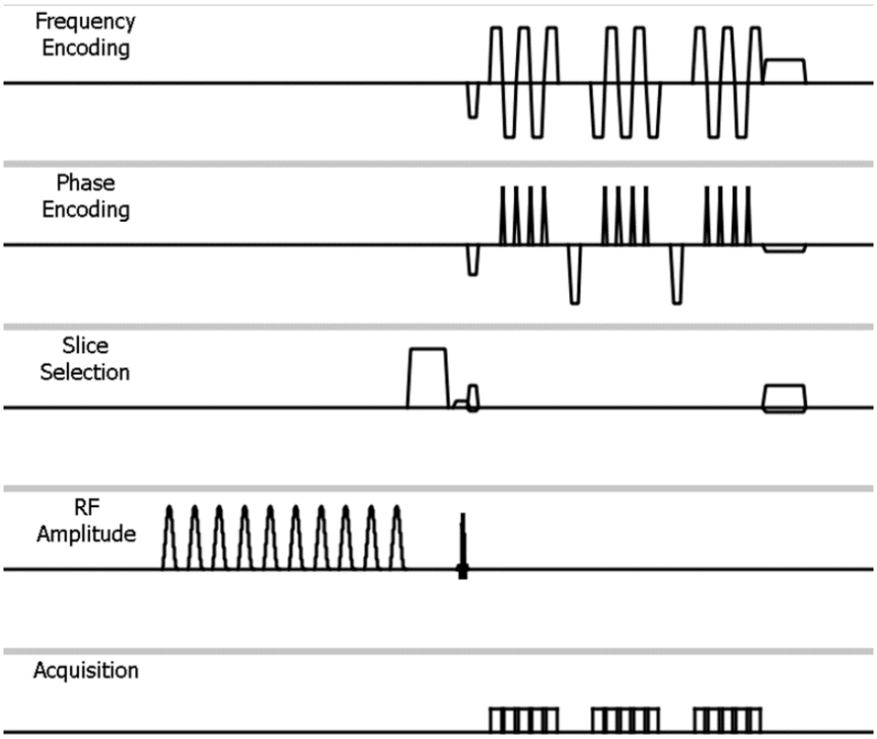

Figure 1 Sequence diagram for one TR

ihMT metrics Here we computed the ihMTdR1sat metric, which is not as widely used nowadays as the ihMTsat metric. However, the ihMT literature is still evolving rapidly and the data acquisition and processing for that project started before most of the literature on ihMTsat was published. The ihMTdR1sat metric as defined in Varma et al., 2015 thus seemed a good choice when looking at metrics that could be extracted from the ihMT data, in addition to the standard ihMTR. The calculation of ihMTdR1sat was performed following the signal derivation introduced in Varma et al., 2015 with one small generalization to allow for a reference image acquired with a different TR (and not only a different flip angle), as regularly performed for MTsat (or ihMTsat) calculations.

Equations of ihMT:

ihMTR was calculated as:

$$ihMTR= (S_{single}-S_{dual})/S_0$$

ihMTdR1sat was calculated according to the following equations (Helms et al. MRM 2008;1396-1407), Varma et al. Proceedings of the ISMRM 2015: 3357)

$$T1_{sat,single}=((S_{single}/\alpha_{MT})-(S_{T1}/\alpha_{T1}))/((S_{T1}*\ \alpha_{T1})/2*TR_{T1}-(S_{single}*\ \alpha_{MT})/2*TR_{MT})$$

$$T1_{sat,dual}=((S_{dual}/\alpha_{MT})-(S_{T1}/\alpha_{T1}))/((S_{T1}*\ \alpha_{T1})/2*TR_{T1}-(S_{dual}*\ \alpha_{MT})/2*TR_{MT})$$

$$ihMTdR1sat=(1/T1_{sat,dual})-(1/T1_{sat,single})$$

where S_{single} is the signal with MT saturation at a single frequency (average of the MT saturation with positive or negative frequency), S_{dual} is the MT signal with alternating frequency saturation, α_{MT} and α_{T1} are the flip angles of the MT-weighted and T1-weighted acquisitions, respectively and TR_{MT} and TR_{T1} are the repetition times of the MT-weighted and T1-weighted acquisitions, respectively. S_0 is the signal without saturation, at the same flip angle and TR as the acquisition with MT saturation.

In addition, when calculating both ihMTdR1sat and ihMTsat from the same dataset, the two metrics show a very high correlation (Figure 2).

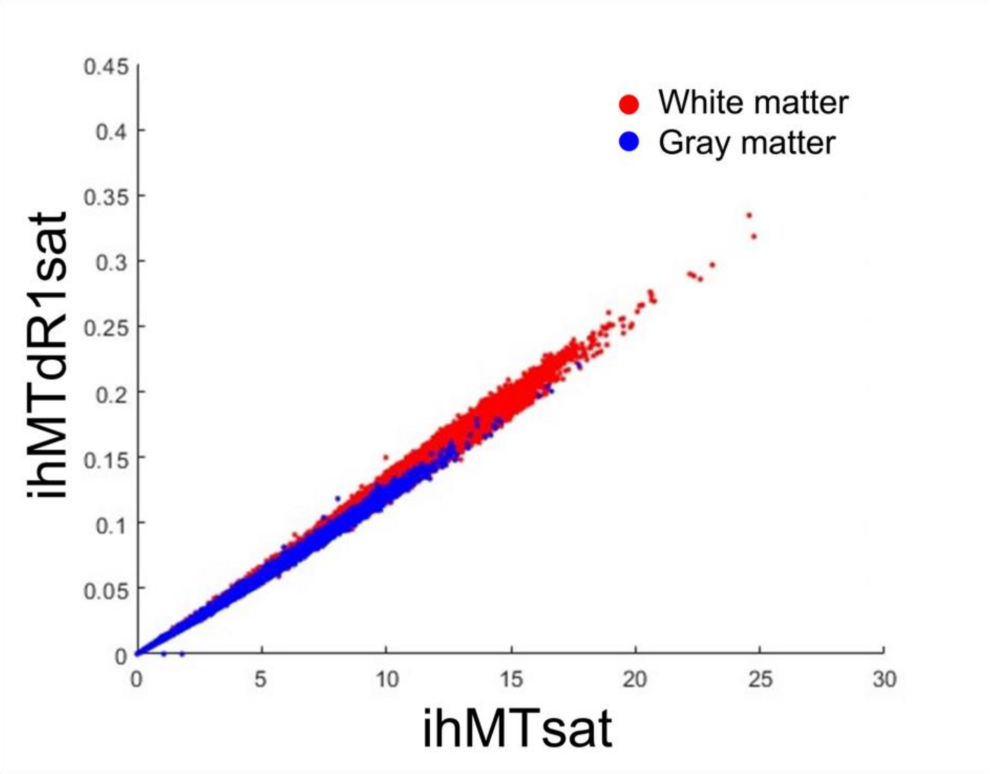

Figure 2 ihMT metrics correlations. Correlation between ihMTsat and ihMTdR1sat computed from a single subject and single visit.

3. Bundle section

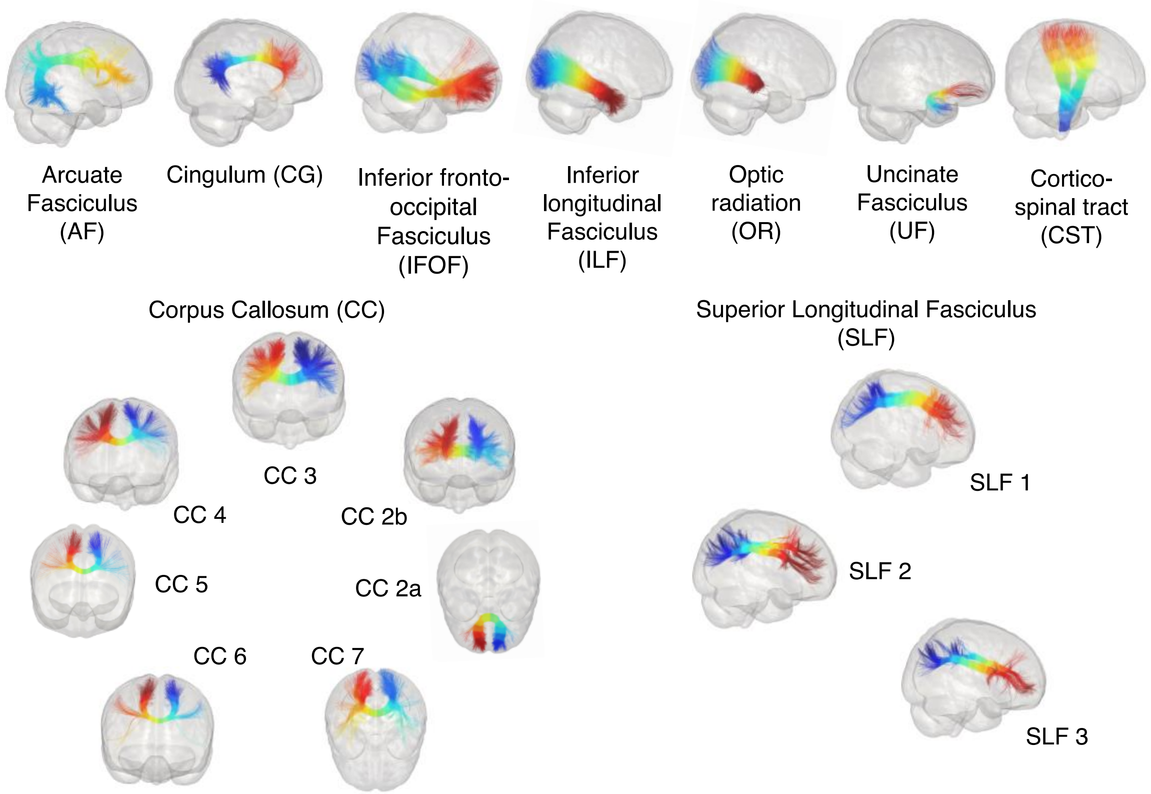

Figure 3 Representation of bundle sections. The major bundle models used by RecobundlesX as shape priors to extract the bundles from the whole tractogram were resampled into 10 segments for illustration. Left and right have been merged. The colors displayed on the bundles represent the section numbers from 1 (blue) to 10 (red).

4. Pipeline analysis overview description

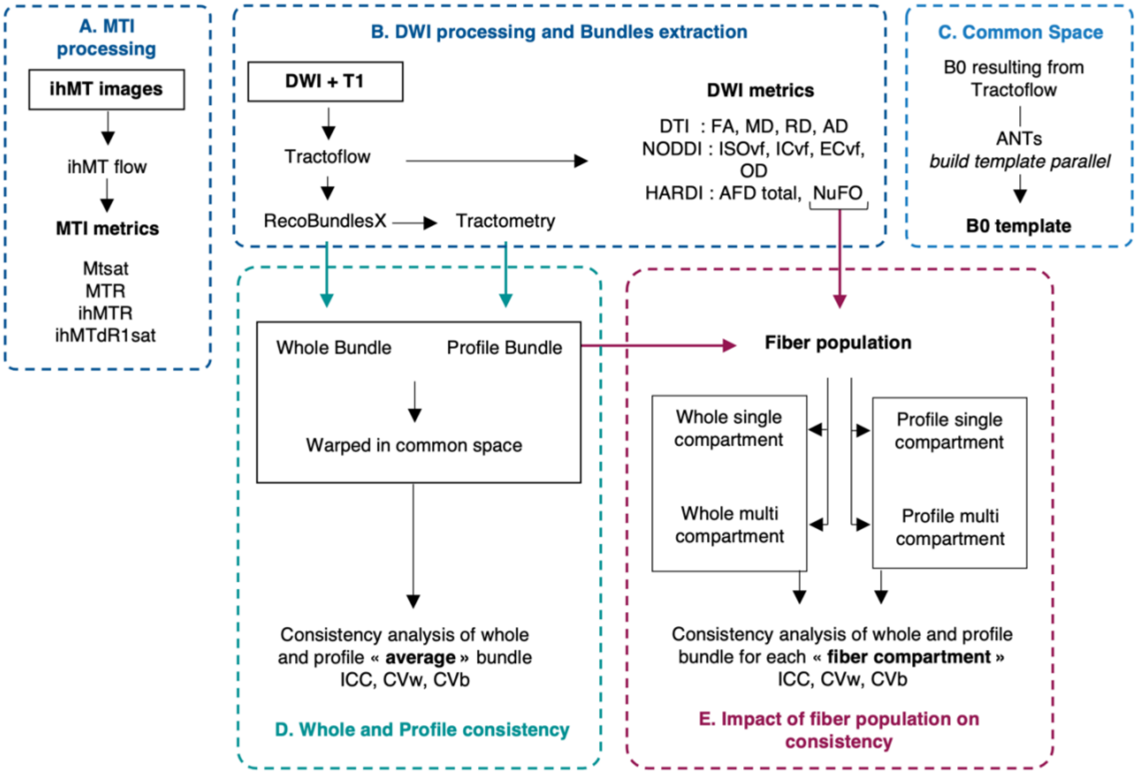

Figure 4 Processes and analyses overview. In A (blue), MTI input and output files using ihMT flow. In B (blue), the DWI process using Tractoflow to generate the tractograms and diffusion measures maps, the bundles' virtual segmentation using RecoBundlesX to obtain whole bundle mask, and bundles resampling using the Tractometry flow which provides bundle profile masks. In C (light blue), diffusion common space generation using ANTs. In D (green), consistency analyses of diffusion and myelin measures from the whole and profile bundle masks. In E (red) processes that take the NuFO map generated in B and the whole and profile bundle mask generated in D to separate them into single and multi compartments masks and perform consistency analyses of diffusion and myelin measures from these masks.

5. Visual quality criteria

The visual quality assessment procedure was applied to the main steps according to the following criteria:

MRI images	*Step*	*Exclusion criteria*
T1, MTI, DWI	**Raw data**	Presence of artifacts that cannot be corrected<br>Incorrect field of view<br>Too high noise in the image<br>Broken image<br>Missing part of the brain (T1, MTI)<br>Incorrect distribution of gradient (DWI)<br>High inhomogeneity (MTI)
T1, MTI, DWI	**Brain extraction**	Eyes included in the brain mask<br>Exclusion of a part of the brain<br>Inclusion of a large part of the background in the brain mask
DWI	**Motion correction**	Alteration of bvecs<br>Remaining motion in the DWI<br>Presence of slice drop (at least in one direction)
DWI	**RGB**	Invalid orientation in major WM structures<br>Low FA value in expected structure (Corpus callosum for example)<br>Global color bias (indicating remaining motion)
T1, MTI, DWI	**Registration**	Poor overlap between warped images and reference image
T1, DWI	**Mask**	Presence of holes in mask<br>Some part of mask missing
DWI	**Bundle segmentation**	Unexpected shape<br>Ends of bundle not in expected locations and/or without expected fanning<br>A low number of streamlines
DWI	**Tract-profile**	Unexpected number of sections<br>Unbalanced sections
MTI, DWI	**Metrics map**	Unexpected range of value (FA > 1 for example)<br>Unexpected range of value in expected structure (low FA value in Corpus callosum for example)

Table 2. Visual quality assessment criteria.

6. Impact of resampling on bundles volume

To ensure that each section of bundles contains enough voxels to assess consistency measurements, we extracted the volume of each section corresponding to the bundle profile analyses. A minimum threshold of 1000 voxels (dotted line) was used to perform analyses, therefore, sections of bundles with fewer than 1000 voxels were excluded. Only the volume of section 10 of the cingulum is below the threshold (circle and red arrow). This section was therefore excluded from the analyses.

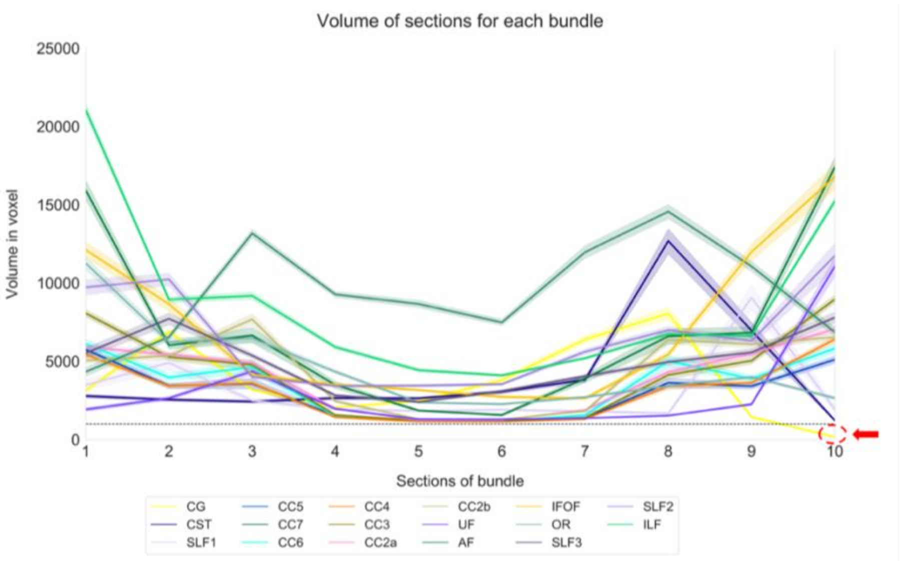

Figure 4 Volume corresponding to each section for each bundle. Colors code for bundles. The volume is expressed in voxels. The black dotted line corresponds to the threshold of 1000 voxels.

7. Impact of ISOvf thresholding on consistency measures

Isotropic Volume Fraction (ISOvf) has generally very low values in the WM and many studies emphasized the poor reliability of this NODDI parameter. To improve the consistency of this parameter, we evaluated the impact of different thresholds to remove values close to zero. A range of thresholds between 0 and 1 with a step size of 0.1 was used. The threshold of 0.045 was chosen because it corresponds relatively to the inflection point of the ICC curve (red dotted line).

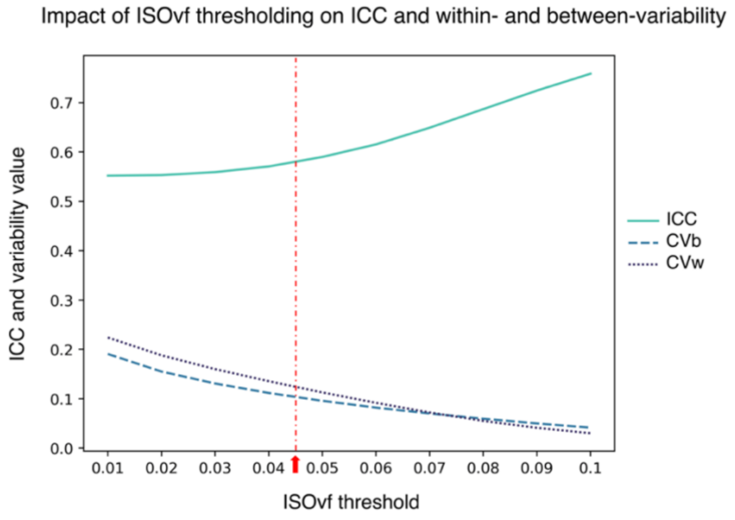

Figure 5 Consistency measures according to ISOvf thresholds. The graph illustrates the ICC (green), within- (dark blue, dotted line) and between-variability (blue, dashed line) values according to the different ISOvf thresholds. The red dotted line represents the chosen threshold of 0.045.
